# Formal mentorship in a surgical residency training program: a prospective interventional study

**DOI:** 10.1186/s40463-017-0186-2

**Published:** 2017-02-13

**Authors:** Han Zhang, Andre Isaac, Erin D. Wright, Yaser Alrajhi, Hadi Seikaly

**Affiliations:** Division of Otolaryngology—Head and Neck Surgery, University of Alberta, Edmonton, Alberta Canada

**Keywords:** Mentorship, Medical learning, Resident stress, Resident burnout

## Abstract

**Background:**

Otolaryngology-Head and Neck surgery resident physicians (OHNSR) have a high prevalence of burnout, job dissatisfaction and stress as shown within the literature. Formal mentorship programs (FMP) have a proven track record of enhancing professional development and academic success. More importantly FMP have an overall positive impact on residents and assist in improving job satisfaction. The purpose of the study is to determine the effects of a FMP on the well-being of OHNSR.

**Methods:**

A FMP was established and all OHNSR participation was voluntary. Eight OHNSR participated in the program. Perceived Stress Survey (PSS) and the Maslach Burnout Inventory (MBI) were administered at baseline and then at 3, 6, 9, and 12 month intervals. World Health Quality of Life-Bref Questionnaire (WH-QOL) was administered at baseline and at 12 months.

**Results:**

Baseline statistics found a significant burden of stress and burnout with an average PSS of 18.5 with a high MBI of 47.6, 50.6, and 16.5 for the emotional, depersonalization, and personal achievement domains respectively. Quality of life was also found to be low with a WH-QOL score of 71.9. After implementation of the FMP, PSS was reduced to 14.5 at 3 months (*p =* 0.174) and a statistically significant lower value of 7.9 at 12 months (*p =* 0.001). Participants were also found to have lower emotional scores (14.9, *p <* 0.0001), levels of depersonalization (20.1, *p <* 0.0001), and higher personal achievement (42.5, *p <* 0.0001) on MBI testing at 12 months. Overall quality values using the WH-QOL was also found to be significantly improved (37.5, *P =* 0.003) with statistically significant lower scores for the physical health (33.9, *p =* 0.003), psychological (41.1, *p =* 0.001), social relationship (46.9, *p =* 0.019), and environment (53.5, *p =* 0.012) domains.

**Conclusion:**

This is the first study to show that FMP can potentially alleviate high levels of stress and burnout within a surgical residency program and achieve higher levels of personal satisfaction as well as overall quality of life.

## Background

Burnout is a maladaptive work-related condition and is characterized by emotional exhaustion, depersonalization, and feeling of lack of personal accomplishment [1]. It is known to affect up to 70% of physicians and residents, often leading to increased levels of stress, depression, job-dissatisfaction, and overall lower quality of life [2–4]. The effects of burnout can be disastrous and many studies have found the extent of such burnout to be alarmingly critical in medical trainees [5].

Many stressors such as daily work demands, caring for sick patients, managing the demands of learning within an environment of job uncertainty are all potential unique contributors to burnouts amongst Otolaryngology-Head and Neck Surgery residents (OHNSR) [6]. Many initiatives such as models for healthy work-life balance and decreasing work hours for residents have been instigated but they do not appear to be successful in mitigating the effects of these complex stressors [7, 8]. A potential approach to counteracting burnout that has not been investigated in the academic setting is mentoring. Effective mentorship can play a critical role in academic success as well as professional growth and development [9]. In addition to the potential of enhancing motivation, and productivity; formal mentoring programs also have an overall positive impact on burnout and may assist in improving job satisfaction as well as career development by providing supports for success [10, 11].

Despite the theoretical benefit of mentorship there is a paucity of programs with a formalized mentorship programs in the current OHNSR training landscape [12–15]. The objective of this project was to create a formalized mentorship program (FMP) within a surgical residency with the goal of meeting the academic as well as the personal needs of the residents. The purpose of this study was to report on the prospective outcomes of this FMP as they pertain to resident well-being and quality of life.

## Methods

Ethics approval was granted by the University of Alberta’s Health Research Ethics Board. Prior to formal mentorship program implementation, residents met with the post-graduate training program director on a bi-annual basis. No formal mentors were assigned to any of the residents. Trainees were encouraged to meet with staff members they were familiar with if any advice was required.

### Formal mentorship program

**Fig. 1:**
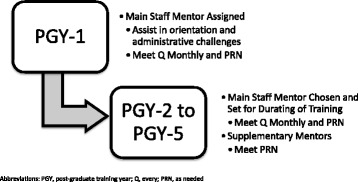
Formal resident mentorship structure

A formal mentorship program was established at the Division of Otolaryngology-Head and Neck Surgery, University of Alberta and the tiered mosaic model of mentorship was chose as the platform. The program was initiated on February 1^st^, 2015. The structure of the program is outlined in Fig. 1. Within this model, each resident is assigned one main mentor and several supplemental mentors. The main mentor serves to facilitate all the areas of guidance including, research, clinical, surgery, or personal development. Supplemental mentors are sought to provide distinct insights into one of the domains because of their distinctive expertise within that area.

Once enrolled within the programs, main mentors are encouraged to meet with their mentees on a monthly basis while supplemental mentors meet with mentees on an as needed basis.

The goals were to create a mentorship program that is beneficial to the academic environment and for the early development of a successful surgical career. We wanted to create a program that met all the demands of the resident and mentors, but was flexible and subject to evolving situations. The program had to have formal structure, good management support, appropriate monitoring, and quality outcome measurements.

### Mentees and mentors

All Post-Graduate Trainees (PGY) from year 1 to year 4 who were enlisted within the University of Alberta, Otolaryngology-Head and Neck Surgery Residency program were invited to participate. All participation was considered voluntary and the residents were able to opt out of the program and or the study at any point. All residents who were invited to participate accepted.

All academic staff within the division were invited to participate in the program as mentors. All 14 academic staff who were invited to participate accepted. However due to a high number of academic staff and a limited number of residents, not all staff served as mentors. An intake form was sent to potential mentors and mentees in order to gauge their personality, areas of expertise, and interest in mentorship. Potential list of mentors were sent to PGY-2 to 4 trainees interested in the FMP. The trainees then choose their own mentors from the list. PGY-1 trainees were matched to a main mentor based on the intake form depending on personality and occupational compatibility. The intake form for the trainee and potential mentors were de-identified and used for matching the PGY-1 trainee and potential mentors by the FMP committee. The PGY-1 mentors assisted in orientation of the new residents and with the administrative challenges of a new environment. At the end of the PGY-1 year, the mentoring relationship was terminated. The resident was then given the choice to seek out a new main mentor or continue with the original assigned mentor. Mentors were encouraged to meet with their mentees every 3 months. Meetings could be formalized or in formalized and could include topics ranging from career planning to personal well-being.

### Governance

A committee was created to help oversee the implementation of the FMP. The committee’s role included conducting yearly evaluations of the main staff to mentee, overseeing issues with mentor to mentee relationships, and most importantly maintaining the integrity and goals of the FMP. The committee at yearly meetings reviewed resident satisfaction as well as burnout and stress data. No formal benchmarks were set for these yearly meetings however worsening or lack of improvement in stress and burnouts were utilized as indications for intervention.

### Outcomes

The primary outcome was the well-being of the OHNSR participating within the FMP. Levels of burnout, stress, and quality of life were measured by the Maslach Burnout Inventory-Human Services (MBI), Perceived Stress Scale (PSS), and the World Health Organization Quality of Life – Bref (WH-QOL) survey at baseline. MBI and PSS were then taken at 3, 6, 9, and 12 month time points. WH-QOL was measured at baseline and at 12 months.

The secondary outcome was the overall satisfaction of the OHNSR residents regarding the FMP assessed using a Likert-style satisfaction scale survey.

### Survey instruments

The MBI-Human Services is a validated and the most widely used instrument to study burnout. It contains 22-items that address 3 domains of burnout: emotional exhaustion, depersonalization, and personal accomplishment [1]. The PSS is a validated 10 item questionnaire used to measure levels of stress [16]. The WH-QOL is a validated 26 item instrument that addresses overall quality of life and 4 other sub-domains: physical health, psychological, social relationships, and environment [17].

### Statistical analysis

Descriptive statistics were used for residents’ parameters. The data was expressed as means. Continuous data was analyzed using analysis of variance (ANOVA). Comparisons of proportions were performed using the chi-squared test. Level significance was set as *p <* 0.05. Analyses were performed with SPSS Statistics 20.0 (SPSS Inc, Chicago, IL).

## Results

Eight residents between PGY-1 to PGY-4 were enrolled in the FMP in February 2015 at the University of Alberta (Edmonton, AB, Canada) and all consented to participating within the study.

### Participant variables

Table 1 demonstrates participant demographic variables. The mean age was found to be 29.1 years (Range: 26–33). Most of the participants were male at 62.5%. In terms of future plans, 37.5% of the residents wanted to pursue an academic career with 50% intending on fellowship training after the conclusion of residency.

**Table 1:** Demographics of participants

Variable	Participants
N	8
Mean Age, yrs (Range)	29.1 (26–33)
Gender, no. (%)	
Males	5 (62.5)
Females	3 (37.5)
Training Level, no. (%)	
PGY-1	2 (25.0)
PGY-2	2 (25.0)
PGY-3	2 (25.0)
PGY-4	2 (25.0)
Career Plan, no. (%)	
Academic Practice	3 (37.5)
Community Practice	2 (25.0)
Unsure	3 (37.5)
Fellowship, no. (%)	
Yes	4 (50.0)
No	0 (0.0)
Unsure	4 (50.0)

*no* number, *PGY* post-graduate training year

### Burnout and stress outcomes

Outcomes of burnout and stress were measured using the MBI and PSS taken at baseline then at 3, 6, 9, and 12 months post-administration of the FMP. All participants had a high level of baseline stress with an average PSS score of 18.5 (Table 2). MBI baseline results showed high average burnout scores of 47.6 and 50.6 in subscales of emotional exhaustion and depersonalization respectively (Table 2). Personal accomplishment was found to be low at 16.5 on the MBI at baseline (Table 2).

**Table 2:** MBI and PSS scores

Survey	Baseline score	3 Months score	6 Months score	9 Months score	12 Months score	*p-*Value (baseline – 12 months)
PSS	18.5	14.5	13.5	8.9	7.9	0.001
MBI						
Emotional Exhaustion	47.6	46.1	35.1	25.5	14.9	<0.0001
Depersonalization	50.6	47.3	35.9	28.5	20.1	<0.0001
Personal Accomplishment	16.5	24.5	30.3	40.1	42.5	<0.0001

*PSS* Perceived Stress Scale, *MBI* Maslach Burnout Inventory-Human Services

**Table 3:** WH-QOL scores

Domains	Baseline score	12 Months score	*p-*Value
Overall QOL	71.9	37.5	0.003
Physical Health	49.6	33.9	0.003
Psychological	57.8	41.1	0.001
Social Relationship	63.5	46.9	0.019
Environment	69.9	53.5	0.012

*WH-QOL* World Health Quality of Life-Bref Questionnaire, *QOL* Quality of Life

**Table 4:** Resident satisfaction Likert-Scale

Components felt benefitted from	Average score
Career Planning	0.85
Research	0.72
Clinical Skills	0.69
Networking	0.76
Collaborations with Colleagues	0.87
Balance between Family and Work	0.63
Knowledge about the Academic System	0.77
Providing a Role Model	0.92
Feel more Supported	0.92
Providing Opportunities	0.86
More Connected to Colleagues	0.90
Increased Visibility	0.79
Having someone to turn to in times of difficulty	0.92
Increased Job Satisfaction	0.81
Increased Learning Satisfaction	0.82
Overall Satisfaction	0.90

Compared to baseline, PSS taken at 3 months showed an average score of 14.5 (*p =* 0.174) and a statistically significant lower level of 7.9 (*p =* 0.001) at 12 months. MBI compared to baseline showed an average score of 46.1, 47.3, and 24.5 for emotional exhaustion, depersonalization, and personal accomplishment at 3 months respectively (*p >* 0.05). At 12 months follow-up, the participants were found to have a statistically significant lower emotional exhaustion at 14.9 (*p <* 0.0001) and level of depersonalization at 20.1 (*p <* 0.0001) along with higher personal achievement at 42.5 (*p <* 0.0001) on MBI.

Table 5 shows the distribution of PSS scores by PGY level. PGY-2 trainees were found to have the highest baseline PSS at 28.5 while PGY-4 trainees were found to have the lowest baselines PSS at 5.5. There was a statistically significant improvement in stress across all the PGY levels (*p <* 0.05) at 1 year after implementation of the FMP.

**Table 5:** PSS scores by Post-Graduate training year

PGY Level	Baseline PSS	Post 1 year FMP PSS	*p-*Value
1	19.5	10.5	0.004
2	28.5	11.5	0.002
3	15.5	9	0.020
4	10.5	5.5	0.025

*PSS* Perceived Stress Scale, *FMP* Formalized Mentorship Program, *PGY* Post-Graduate Training Year

Table 6 shows the distribution of MBI scores by PGY level. PGY-2 trainees much like for PSS were found to have the highest baseline MBI scores for the emotional exhaustion (58) and depersonalization (71) domain as well as the lowest score for the personal accomplishment (26) domain. PGY-4 trainees were found have the lowest baseline MBI across all the domains. There was a statistically significant improvement in burnout across all the PGY levels (*p <* 0.05) at 1 year after implementation of the FMP.

**Table 6:** MBI scores by Post-Graduate training year

PGY Level	Baseline MBI		Post 1 year FMP MBI	*p-*Value
1	EE	44.5	14.0	<0.0001
	DEP	36.5	17.0	<0.0001
	PA	14.0	38.0	<0.0001
2	EE	58	19.5	<0.0001
	DEP	71	30.5	<0.0001
	PA	26	59	0.0002
3	EE	44.5	14.5	<0.0001
	DEP	45	19	<0.0001
	PA	15.5	39.5	<0.0001
4	EE	38	17	0.0002
	DEP	32	21	0.0020
	PA	16	27	0.0040

*MBI* Maslach Burnout Inventory, *EE* emotional exhaustion, *DEP* depersonalization, *PA* personal accomplishment, *FMP* Formalized Mentorship Program, *PGY* Post-Graduate Training Year

### Quality of life outcome

Overall baseline average WH-QOL was found to be 71.9, corresponding to a low quality of life (Table 3). The environment domain was found to yield the lowest score at 69.9. At 12 months post introduction of the FMP, overall average WH-QOL was found to be significantly improved at 37.5 (*p =* 0.003). There was statistically significant improvement in all domains of the WH-QOL questionnaire including the environment domain at 53.5 (*p <* 0.05).

### Participant satisfaction

Overall satisfaction for the FMP was found to be 0.90 on the Likert-scale with the highest scores for “providing a role model”, “feel more supported”, and “having someone to turn to in times of difficult” at 0.92 as shown in Table 4. The FMP program was found to least benefit “clinical skills” and “balance between family and work” at 0.69 and 0.63 respectively.

## Discussion

The word mentor first appeared in the English language in 1616 and is derived from the ancient Greek mythological character synonymous with the word [18]. Mentor, was entrusted by Odysseus for the care and upbringing of his son Telemachus when he parted from his wife Penelope to sail off to the Trojan War [19]. From henceforth, mentor has been cited as “role model, sponsor, and friend to a less skilled or less experienced person for the purpose of promoting the latter’s professional and/or personal development” [20].

The medical definition of mentorship is distinct in that it stresses the emphatic traits of the mentors and distinguishes them from role models [21]. A mentor, then in the medical education sense, is more than a teacher, coach, councilor, or preceptor who provides bits of knowledge to the learner. Mentorship differs in that the mentor is actively engaged in a two-way relationship with the junior colleague, a relationship that evolves and develops over time [22]. An effective mentor, therefore, serves as the guardian and promoter of the young physician’s personal and professional development and takes a personal interest in the success of the mentee [23]. Therefore, the development of a FMP is an integral and essential part of the development of young academic OHNS surgeons, but is often undervalued and overlooked by academic institutions [15].

Mentorship is a process with distinct structural, interactional, and temporal features. Structurally, it can be delivered through multiple models. The tiered mosaic model used within our program combines the traditional dyadic model mentorship by having a main mentor and supplemental advisors provided by multiple mentors with distinct accomplishments in areas such as research, clinical, surgery, or personal development [24]. Utilizing this model of mentorship allows the mentee to benefit from their main mentor’s experience and relationship while overcoming the shortcomings of the traditional dyadic model of mentoring where one individual is expected to meet all the needs of the mentee. Due to these advantages and the impracticality of the dyadic model of mentorship in today’s academic environment, the tiered mosaic model of mentorship was chosen [25].

Many studies have shown that medical residency training is a stressful endeavor [2–5, 8, 26, 27]. The demands of balancing a hectic life of caring for sick patients with personal life as well as meeting learning objectives often lead to high amounts of stress culminating eventually in burnout. Our group of residents had a high baseline overall WH-QOL score of 71.9 as well as scores of 49.6, 57.8, 63.5, 69.9 for the domains of physical health, psychological health, social relationship, and environment respectively. MBI scores for emotional exhaustion (47.6), depersonalization (50.6), and low personal accomplishment (16.5) were also found to be within the upper third of normal. These baseline MBI scores were found to be highest amongst PGY-2 trainees. While at first glance these results seem alarming, other studies within the medical resident population have shown similar and often times higher levels of burnout. Dolittle et al. showed amongst their cohort of internal medicine resident’s extremely high baseline levels of emotional exhaustion (94), depersonalization (97), and low personal accomplishment (9), all of which correspond with the upper third of the normal value in the MBI [26]. These results were also further reinforced by Bellieni et al., where a cohort of neonatologists exhibited a similarly high level of MBI domains [5].

FMP has been shown in the literature to be an integral and essential part of academic medicine with benefits of work productivity as well as personal well-being and job satisfaction [15, 21, 23, 25]. Within our own cohort of residents, the FMP has proven to have made a remarkable difference decreasing stress and burnout while improving quality of life. Initial results of the program showed a trend towards improvement in MBI with a score of 46.1, 47.3, and 24.5 for subcategories of emotional exhaustion, depersonalization, and personal accomplishment at 3 months (*p >* 0.05). Similarity at the same time interval, PSS was found to be improved without reaching statistical significance (14.5, *p =* 0.174). Survey results at 6 month follow-up however yielded statistically significant enhanced results for MBI for emotional exhaustion (35.1, *p <* 0.05), depersonalization (35.9, *p <* 0.05), and personal accomplishment (30.3, *p <* 0.05). Improvements continued up to 12 months where MBI reached the lower third percentile of normal for all three subcategories. Similar results were found in PSS where a statistically significant improvement was found up to 12 months (7.9, *p =* 0.001). The improvements in PSS and MBI were found to be statistically significant amongst all the PGY levels as shown in Table 5 and 6 (*p <* 0.05). WH-QOL survey also showed a statistically significant improvement at 12 months (37.5, *P =* 0.003). The lack of significant progresses early on could be attributed to several factors. Instituting a new formalized program is often accompanied by a level of learning and development. Logistic as well as organization issues likely contributed to a potential delay in residents matching to their potential mentors as well as a period of learning and adapting to the new system. In addition, the intimate mentorship relationships often require comfort, commitment, and interpersonal chemistry; all factors that require time to build and foster.

While the improvements in the quality of life of our cohort of residents within a relative short period of time are encouraging, the role of the academic learning environment independent of the FMP cannot be discounted. This study was designed as a prospective interventional study without controls. As a result, it is susceptible to confounders that may influence the overall improvements such as personal well-being, stress, and burnout which are factors of multi-factorial origin within each individual person. Trainees are also potentially subject to possible bias from the Hawthorne effect which cannot be excluded owing to the design of the study [28]. In addition, residents are immersed in a unique and complex environment encompassing patient care, medical learning, and personal well-being. Factors that may influence any part of a resident’s life is certain to make a significant impact on the MBI, WH-QOL, as well as PSS scores. We cannot completely quantify the effect of the FMP on the improvements in our cohort of residents but we can verify that within the 12 month period of the study, there were no other major changes in the structure of the residency training program or learning environment other than the FMP instigation. Therefore, while no conclusive direct cause and effect relationship can be ascertained between the FMP and the improvements of resident well-being, the study does provide external validity on the need of the FMP in an OHNS residency program. It also provides a potential valuable tool in treating the high levels of stress and burnout within the surgical trainee cohort.

Utilization of FMP within the current medical residency training landscape is still in its infancy. As we move towards more formalized, structured, and competency based learning regimes, the critical role of a FMP cannot be overstated in professional growth and development as well as alleviating stress and preventing burnout. While more multi-institutional prospective studies would provide more definitive evidence on the magnitude of the true benefit of FMP, this study provides compelling evidence of the critical role FMP plays in an academic surgical residency training program.

## Conclusion

To our knowledge, this is the first study to show that FMP can potentially help alleviate high levels of stress and burnout within a surgical residency program and achieve higher levels of personal satisfaction as well as overall quality of life in OHNS residents.

## References

[1] Maslach C, Jackson SE, Leiter MP (1996). Maslach burnout inventory manual.

[2] Lloyd S, Streiner D, Shannon S (1994). Burnout, depression, life and job satisfaction among Canadian emergency physicians. J Emerg Med.

[3] Spickard A, Gabbe SG, Christensen JF (2002). Mid-career burnout in generalist and specialist physicians. JAMA.

[4] Thomas NK (2004). Resident burnout. JAMA.

[5] Bellieni CV, Righetti P, Ciampa R, Iacoponi F, Coviello C, Buonocore G (2012). Assessing burnout among neonatologists. J Matern Fetal Neonatal Med.

[6] Brandt MG, Scott GM, Doyle PC, Ballagh RH (2014). Otolaryngology - head and neck surgeon unemployment in Canada: a cross-sectional survey of graduating otolaryngology - head and neck surgery residents. J Otolaryngol Head Neck Surg.

[7] Martini S, Arfken CL, Balon R (2006). Comparison of burnout among medical residents before and after the implementation of work hours limits. Acad Psychiatry.

[8] Rosen IM, Gimotty PA, Shea JA, Bellini LM (2006). Evolution of sleep quantity, sleep deprivation, mood disturbances, empathy, and burnout among interns. Acad Med.

[9] Jackson VA, Palepu A, Szalacha L, Caswell C, Carr PL, Inui T (2003). “Having the right chemistry”: a qualitative study of mentoring in academic medicine. Acad Med.

[10] Lee C, Carmen ME (2011). The correlation of mentoring and Job satisfaction: a pilot study of mental health professionals. Community Ment Health J.

[11] Illes J, Glover GH, Wexler L, Leung AN, Glazer GM (2000). A model for faculty mentoring in academic radiology. Acad Radiol.

[12] Gurgel RK, Schiff BA, Flint JH (2010). Mentoring in otolaryngology training programs. Otolaryngol Head Neck Surg.

[13] Hsu AK, Tabaee A, Persky MS (2010). Mentorship in otolaryngology residency: the resident perspective. Laryngoscope.

[14] Zahtz G, Vambutas A, Hussey HM, Rosen L (2014). Resident research experience and career path association: a national survey of recent otolaryngology graduates. Otolaryngol Head Neck Surg.

[15] Sambunjak D, Straus SE, Marusic A (2006). Mentoring in academic medicine: a systematic review. JAMA.

[16] Taylor JM (2015). Psychometric analysis of the Ten-item perceived stress scale. Psychol Assess.

[17] Su CT, Ng HS, Yang AL, Lin CY (2014). Psychometric evaluation of the short form 36 health survey (SF-36) and the world health organization quality of life scale brief version (WHOQOL-BREF) for patients with schizophrenia. Psychol Assess.

[18] Mentor. In Oxford dictionary online. 2014. Accessed 1 Apr 2016.

[19] Zusan E, Vaughan A, Welling RE (2006). Mentorship in a community-based residency program. Am Surg.

[20] Anderson EM, Shannon AL (1988). Toward a conceptualization of mentoring. J Teach Educ.

[21] Healy NA, Cantillon P, Malone C, Kerin MJ (2012). Role models and mentors in surgery. Am J Surg.

[22] Paice E, Heard S, Moss F (2002). How important are role models in making good doctors?. BMJ.

[23] Pellegrini VD (2006). Mentoring during residency education: a unique challenge for the surgeon?. Clin Orthop Relat Res.

[24] Morahan PS, Richman RC (2001). Career obstacles for women in medicine. Med Educ.

[25] Patel VM, Warren O, Ahmed K (2011). How can we build mentorship in surgeons of the future?. ANZ J Surg.

[26] Doolittle BR, Windish DM, Seelig CB (2013). Burnout, coping, and spirituality among internal medicine resident physicians. J Grad Med Educ.

[27] Fletcher AM, Pagedar N, Smith RJ (2012). Factors correlating with burnout in practicing otolaryngologists. Otolaryngol Head Neck Surg.

[28] Ulmer FC (1976). The Hawthorne effect. Educ Dir Dent Aux.

